# 2-(4-Bromo­phen­yl)-2-oxoethyl 2-methyl­benzoate

**DOI:** 10.1107/S1600536811044564

**Published:** 2011-10-29

**Authors:** Hoong-Kun Fun, Chin Wei Ooi, B. Garudachari, Arun M. Isloor, M. N. Satyanarayan

**Affiliations:** a X-ray Crystallography Unit, School of Physics, Universiti Sains Malaysia, 11800 USM, Penang, Malaysia; b Organic Electronics Division, Department of Chemistry, National Institute of Technology-Karnataka, Surathkal, Mangalore 575 025, India; c Department of Physics, National Institute of Technology-Karnataka, Surathkal, Mangalore 575 025, India

## Abstract

In the title compound, C_16_H_13_BrO_3_, the dihedral angle formed between the bromo- and methyl-substituted benzene rings is 66.66 (8)°. In the crystal, mol­ecules are linked by inter­molecular C—H⋯O hydrogen bonds, forming a two-dimensional network parallel to the *ac* plane. The crystal packing is further consolidated by C—H⋯π inter­actions.

## Related literature

For background and applications of phenacyl benzoates, see: Rather & Reid (1919 ▶); Sheehan & Umezaw (1973 ▶); Ruzicka *et al.* (2002 ▶); Litera *et al.* (2006 ▶); Huang *et al.* (1996 ▶); Gandhi *et al.* (1995 ▶). For a related structure, see: Fun *et al.* (2011 ▶). For the synthesis, see: Judefind & Reid (1920 ▶). For bond-length data, see: Allen *et al.* (1987 ▶).
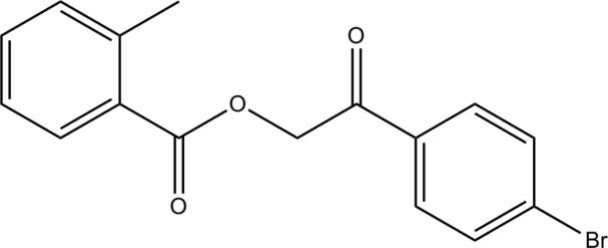


## Experimental

### 

#### Crystal data

C_16_H_13_BrO_3_
*M* *_r_* = 333.17
Monoclinic, 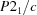

*a* = 5.4519 (1) Å
*b* = 31.2382 (5) Å
*c* = 9.7206 (1) Å
β = 120.410 (1)°
*V* = 1427.74 (4) Å^3^
*Z* = 4
Mo *K*α radiation
μ = 2.88 mm^−1^
*T* = 100 K
0.51 × 0.36 × 0.08 mm

#### Data collection

Bruker SMART APEXII CCD area-detector diffractometer
Absorption correction: multi-scan (*SADABS*; Bruker, 2009 ▶) *T*_min_ = 0.323, *T*_max_ = 0.811
20164 measured reflections
5211 independent reflections
4181 reflections with *I* > 2σ(*I*)
*R*_int_ = 0.032

#### Refinement

*R*[*F* ^2^ > 2σ(*F* ^2^)] = 0.040
*wR*(*F* ^2^) = 0.094
*S* = 1.04
5211 reflections
182 parameters
H-atom parameters constrained
Δρ_max_ = 0.85 e Å^−3^
Δρ_min_ = −0.43 e Å^−3^

### 

Data collection: *APEX2* (Bruker, 2009 ▶); cell refinement: *SAINT* (Bruker, 2009 ▶); data reduction: *SAINT*; program(s) used to solve structure: *SHELXTL* (Sheldrick, 2008 ▶); program(s) used to refine structure: *SHELXTL*; molecular graphics: *SHELXTL*; software used to prepare material for publication: *SHELXTL* and *PLATON* (Spek, 2009 ▶).

## Supplementary Material

Crystal structure: contains datablock(s) global, I. DOI: 10.1107/S1600536811044564/is2797sup1.cif

Structure factors: contains datablock(s) I. DOI: 10.1107/S1600536811044564/is2797Isup2.hkl

Supplementary material file. DOI: 10.1107/S1600536811044564/is2797Isup3.cml

Additional supplementary materials:  crystallographic information; 3D view; checkCIF report

## Figures and Tables

**Table 1:** Hydrogen-bond geometry (Å, °) *Cg*1 and *Cg*2 are the centroids of the C1–C6 and C10–C15 rings, respectively.

*D*—H⋯*A*	*D*—H	H⋯*A*	*D*⋯*A*	*D*—H⋯*A*
C8—H8*A*⋯O2^i^	0.99	2.32	3.224 (2)	151
C8—H8*B*⋯O2^ii^	0.99	2.52	3.447 (3)	156
C15—H15*A*⋯*Cg*1^iii^	0.95	2.74	3.5472 (19)	143
C16—H16*B*⋯*Cg*1^iv^	0.98	2.98	3.4909 (19)	114
C2—H2*A*⋯*Cg*2^v^	0.95	2.91	3.5915 (19)	130

Symmetry codes: (i) 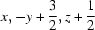; (ii) 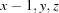; (iii) 
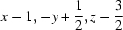
; (iv) 
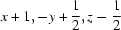
; (v) 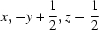.

**Fig. 1.**
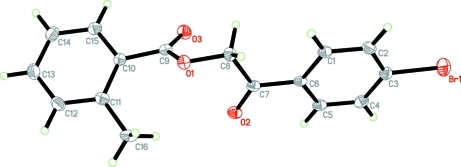
The molecular structure of the title compound, showing 50% probability displacement ellipsoids.

**Fig. 2.**
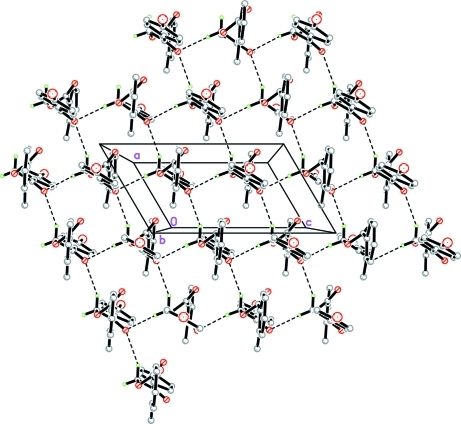
The crystal packing of the title compound. The dashed lines represent the hydrogen bonds.

## supplementary crystallographic information

### Comment

Phenacyl benzoate derivatives are very important in identification of organic acids (Rather & Reid, 1919), since they undergo photolysis in neutral and mild conditions (Sheehan & Umezaw, 1973; Ruzicka *et al.*, 2002; Litera *et al.*, 2006). They find applications in the field of synthetic chemistry for the synthesis of oxazoles, imidazoles (Huang *et al.*, 1996) and benzoxazepine (Gandhi *et al.*, 1995). We hereby report the crystal structure of 2-(4-bromophenyl)-2-oxoethyl 2-methylbenzoate which has potential commercial importance.

In the title compound (Fig. 1), the dihedral angle formed between the bromo-substituted (C1–C6) and the methyl-substituted (C10–C15) benzene rings is 66.66 (8)°. The bond lengths (Allen *et al.*, 1987) and angles are within normal ranges and are comparable to the related structure (Fun *et al.*, 2011).

In the crystal packing (Fig. 2), the molecules are linked by intermolecular C8—H8A···O2 and C8—H8B···O2 hydrogen bonds (Table 1), forming a two-dimensional network parallel to the *ac* plane. The crystal packing is further consolidated by C—H···π interactions, involving the centroids of the bromo-substituted (C1–C6; *Cg*1; Table 1) and methyl-substituted benzene rings (C10–C15; *Cg*2; Table 1).

### Experimental

The mixture of 2-methylbenzoic acid (1.0 g, 0.0073 mol), potassium carbonate (1.10 g, 0.0080 mol) and 2-bromo-1-(4-bromophenyl)ethanone (2.02 g, 0.0073 mol) in dimethylformamide (10 ml) was stirred at room temperature for 2 h. On cooling, colourless needle-shaped crystals of 2-(4-bromophenyl)-2-oxoethyl 2-methylbenzoate began to separate out. It was collected by filtration and recrystallized from ethanol. Yield: 2.35 g, 96.3%. *M.p.*: 330–331 K (Judefind & Reid, 1920).

### Refinement

All the H atoms were positioned geometrically (C—H = 0.95, 0.98 or 0.99 Å) and refined using a riding model, with *U*_iso_(H) = 1.2 or 1.5*U*_eq_(C). A rotating group model was applied to the methyl group. In the final refinement, one outliner (0 2 0) was omitted.

### Crystal data

**Table:**

C_16_H_13_BrO_3_	*F*(000) = 672
*M**_r_* = 333.17	*D*_x_ = 1.550 Mg m^−^^3^
Monoclinic, *P*2_1_/*c*	Mo *K*α radiation, λ = 0.71073 Å
Hall symbol: -P 2ybc	Cell parameters from 7724 reflections
*a* = 5.4519 (1) Å	θ = 2.6–32.6°
*b* = 31.2382 (5) Å	µ = 2.88 mm^−^^1^
*c* = 9.7206 (1) Å	*T* = 100 K
β = 120.410 (1)°	Plate, colourless
*V* = 1427.74 (4) Å^3^	0.51 × 0.36 × 0.08 mm
*Z* = 4	

### Data collection

**Table:**

Bruker SMART APEXII CCD area-detector diffractometer	5211 independent reflections
Radiation source: fine-focus sealed tube	4181 reflections with *I* > 2σ(*I*)
graphite	*R*_int_ = 0.032
φ and ω scans	θ_max_ = 32.7°, θ_min_ = 2.5°
Absorption correction: multi-scan (*SADABS*; Bruker, 2009)	*h* = −8→8
*T*_min_ = 0.323, *T*_max_ = 0.811	*k* = −35→47
20164 measured reflections	*l* = −14→14

### Refinement

**Table:**

Refinement on *F*^2^	Primary atom site location: structure-invariant direct methods
Least-squares matrix: full	Secondary atom site location: difference Fourier map
*R*[*F*^2^ > 2σ(*F*^2^)] = 0.040	Hydrogen site location: inferred from neighbouring sites
*wR*(*F*^2^) = 0.094	H-atom parameters constrained
*S* = 1.04	*w* = 1/[σ^2^(*F*_o_^2^) + (0.0391*P*)^2^ + 0.9854*P*] where *P* = (*F*_o_^2^ + 2*F*_c_^2^)/3
5211 reflections	(Δ/σ)_max_ = 0.003
182 parameters	Δρ_max_ = 0.85 e Å^−^^3^
0 restraints	Δρ_min_ = −0.43 e Å^−^^3^

### Special details

**Table:**

Experimental. The crystal was placed in the cold stream of an Oxford Cryosystems Cobra open-flow nitrogen cryostat (Cosier & Glazer, 1986) operating at 100.0 (1) K.
Geometry. All e.s.d.'s (except the e.s.d. in the dihedral angle between two l.s. planes) are estimated using the full covariance matrix. The cell e.s.d.'s are taken into account individually in the estimation of e.s.d.'s in distances, angles and torsion angles; correlations between e.s.d.'s in cell parameters are only used when they are defined by crystal symmetry. An approximate (isotropic) treatment of cell e.s.d.'s is used for estimating e.s.d.'s involving l.s. planes.
Refinement. Refinement of *F*^2^ against ALL reflections. The weighted *R*-factor *wR* and goodness of fit *S* are based on *F*^2^, conventional *R*-factors *R* are based on *F*, with *F* set to zero for negative *F*^2^. The threshold expression of *F*^2^ > σ(*F*^2^) is used only for calculating *R*-factors(gt) *etc*. and is not relevant to the choice of reflections for refinement. *R*-factors based on *F*^2^ are statistically about twice as large as those based on *F*, and *R*- factors based on ALL data will be even larger.

### Fractional atomic coordinates and isotropic or equivalent isotropic displacement parameters (Å^2^)

**Table:**

	*x*	*y*	*z*	*U*_iso_*/*U*_eq_	
Br1	0.17278 (4)	0.970131 (6)	0.70447 (3)	0.03247 (7)	
O1	0.2143 (2)	0.70425 (4)	0.82251 (14)	0.0169 (2)	
O2	0.4582 (2)	0.75657 (4)	0.70793 (14)	0.0185 (2)	
O3	−0.1113 (3)	0.70407 (4)	0.56018 (15)	0.0219 (3)	
C1	0.1466 (3)	0.84316 (5)	0.81729 (19)	0.0167 (3)	
H1A	0.0726	0.8273	0.8715	0.020*	
C2	0.1160 (3)	0.88749 (6)	0.8057 (2)	0.0194 (3)	
H2A	0.0232	0.9020	0.8526	0.023*	
C3	0.2231 (3)	0.91002 (6)	0.7248 (2)	0.0197 (3)	
C4	0.3636 (4)	0.88967 (6)	0.6568 (2)	0.0202 (3)	
H4A	0.4370	0.9057	0.6024	0.024*	
C5	0.3940 (3)	0.84555 (6)	0.6701 (2)	0.0177 (3)	
H5A	0.4897	0.8312	0.6246	0.021*	
C6	0.2854 (3)	0.82191 (5)	0.74968 (18)	0.0145 (3)	
C7	0.3187 (3)	0.77446 (5)	0.75677 (18)	0.0147 (3)	
C8	0.1688 (4)	0.74932 (5)	0.8257 (2)	0.0170 (3)	
H8A	0.2405	0.7585	0.9371	0.020*	
H8B	−0.0376	0.7554	0.7635	0.020*	
C9	0.0627 (3)	0.68539 (5)	0.67839 (19)	0.0156 (3)	
C10	0.1268 (3)	0.63882 (5)	0.68481 (19)	0.0154 (3)	
C11	0.4045 (3)	0.62214 (6)	0.7696 (2)	0.0178 (3)	
C12	0.4349 (4)	0.57773 (6)	0.7654 (2)	0.0234 (3)	
H12A	0.6203	0.5657	0.8198	0.028*	
C13	0.2030 (4)	0.55090 (6)	0.6846 (2)	0.0259 (4)	
H13A	0.2306	0.5208	0.6866	0.031*	
C14	−0.0708 (4)	0.56778 (6)	0.6002 (2)	0.0239 (3)	
H14A	−0.2306	0.5495	0.5440	0.029*	
C15	−0.1062 (3)	0.61174 (6)	0.5995 (2)	0.0191 (3)	
H15A	−0.2917	0.6236	0.5400	0.023*	
C16	0.6654 (3)	0.64980 (6)	0.8579 (2)	0.0223 (3)	
H16A	0.8327	0.6338	0.8739	0.033*	
H16B	0.6905	0.6579	0.9616	0.033*	
H16C	0.6428	0.6757	0.7954	0.033*	

### Atomic displacement parameters (Å^2^)

**Table:**

	*U*^11^	*U*^22^	*U*^33^	*U*^12^	*U*^13^	*U*^23^
Br1	0.03491 (11)	0.01766 (9)	0.04329 (13)	0.00216 (7)	0.01866 (9)	0.00472 (8)
O1	0.0187 (5)	0.0162 (5)	0.0144 (5)	0.0007 (4)	0.0074 (4)	−0.0001 (4)
O2	0.0168 (5)	0.0230 (6)	0.0175 (6)	0.0007 (4)	0.0101 (4)	−0.0028 (4)
O3	0.0182 (5)	0.0211 (6)	0.0189 (6)	0.0023 (4)	0.0038 (5)	0.0018 (5)
C1	0.0155 (7)	0.0202 (7)	0.0153 (7)	−0.0010 (6)	0.0085 (6)	−0.0001 (6)
C2	0.0162 (7)	0.0205 (8)	0.0207 (8)	0.0020 (6)	0.0086 (6)	−0.0017 (6)
C3	0.0161 (7)	0.0180 (7)	0.0214 (8)	−0.0003 (6)	0.0068 (6)	0.0011 (6)
C4	0.0191 (7)	0.0228 (8)	0.0190 (8)	−0.0032 (6)	0.0099 (6)	0.0020 (6)
C5	0.0149 (7)	0.0244 (8)	0.0157 (7)	−0.0013 (6)	0.0090 (6)	−0.0012 (6)
C6	0.0106 (6)	0.0193 (7)	0.0117 (6)	−0.0006 (5)	0.0044 (5)	−0.0008 (5)
C7	0.0111 (6)	0.0203 (7)	0.0094 (6)	−0.0006 (5)	0.0028 (5)	−0.0020 (5)
C8	0.0210 (7)	0.0163 (7)	0.0168 (7)	−0.0002 (6)	0.0118 (6)	−0.0016 (6)
C9	0.0135 (6)	0.0182 (7)	0.0151 (7)	−0.0016 (5)	0.0073 (5)	−0.0007 (5)
C10	0.0163 (7)	0.0168 (7)	0.0139 (7)	0.0002 (5)	0.0081 (6)	0.0007 (5)
C11	0.0173 (7)	0.0226 (8)	0.0154 (7)	0.0020 (6)	0.0096 (6)	0.0026 (6)
C12	0.0226 (8)	0.0229 (8)	0.0282 (9)	0.0072 (6)	0.0155 (7)	0.0058 (7)
C13	0.0307 (9)	0.0178 (8)	0.0343 (10)	0.0040 (7)	0.0203 (8)	0.0044 (7)
C14	0.0258 (8)	0.0201 (8)	0.0280 (9)	−0.0044 (7)	0.0152 (7)	−0.0018 (7)
C15	0.0168 (7)	0.0211 (8)	0.0185 (8)	−0.0005 (6)	0.0082 (6)	0.0000 (6)
C16	0.0140 (7)	0.0303 (9)	0.0204 (8)	0.0018 (6)	0.0072 (6)	−0.0009 (7)

### Geometric parameters (Å, °)

**Table:**

Br1—C3	1.8935 (18)	C8—H8A	0.9900
O1—C9	1.3493 (19)	C8—H8B	0.9900
O1—C8	1.433 (2)	C9—C10	1.490 (2)
O2—C7	1.2170 (19)	C10—C15	1.397 (2)
O3—C9	1.207 (2)	C10—C11	1.407 (2)
C1—C2	1.392 (2)	C11—C12	1.400 (2)
C1—C6	1.396 (2)	C11—C16	1.507 (2)
C1—H1A	0.9500	C12—C13	1.383 (3)
C2—C3	1.386 (2)	C12—H12A	0.9500
C2—H2A	0.9500	C13—C14	1.393 (3)
C3—C4	1.393 (2)	C13—H13A	0.9500
C4—C5	1.386 (2)	C14—C15	1.386 (2)
C4—H4A	0.9500	C14—H14A	0.9500
C5—C6	1.399 (2)	C15—H15A	0.9500
C5—H5A	0.9500	C16—H16A	0.9800
C6—C7	1.491 (2)	C16—H16B	0.9800
C7—C8	1.512 (2)	C16—H16C	0.9800
			
C9—O1—C8	115.46 (13)	O3—C9—O1	123.39 (15)
C2—C1—C6	120.43 (15)	O3—C9—C10	124.51 (15)
C2—C1—H1A	119.8	O1—C9—C10	112.05 (13)
C6—C1—H1A	119.8	C15—C10—C11	120.62 (15)
C3—C2—C1	118.88 (15)	C15—C10—C9	116.27 (14)
C3—C2—H2A	120.6	C11—C10—C9	123.10 (14)
C1—C2—H2A	120.6	C12—C11—C10	117.24 (15)
C2—C3—C4	121.92 (16)	C12—C11—C16	119.55 (15)
C2—C3—Br1	118.84 (13)	C10—C11—C16	123.17 (15)
C4—C3—Br1	119.24 (13)	C13—C12—C11	121.96 (16)
C5—C4—C3	118.51 (15)	C13—C12—H12A	119.0
C5—C4—H4A	120.7	C11—C12—H12A	119.0
C3—C4—H4A	120.7	C12—C13—C14	120.28 (17)
C4—C5—C6	120.84 (15)	C12—C13—H13A	119.9
C4—C5—H5A	119.6	C14—C13—H13A	119.9
C6—C5—H5A	119.6	C15—C14—C13	118.89 (17)
C1—C6—C5	119.42 (15)	C15—C14—H14A	120.6
C1—C6—C7	122.24 (14)	C13—C14—H14A	120.6
C5—C6—C7	118.33 (14)	C14—C15—C10	120.96 (16)
O2—C7—C6	121.60 (15)	C14—C15—H15A	119.5
O2—C7—C8	121.25 (15)	C10—C15—H15A	119.5
C6—C7—C8	117.14 (13)	C11—C16—H16A	109.5
O1—C8—C7	111.25 (13)	C11—C16—H16B	109.5
O1—C8—H8A	109.4	H16A—C16—H16B	109.5
C7—C8—H8A	109.4	C11—C16—H16C	109.5
O1—C8—H8B	109.4	H16A—C16—H16C	109.5
C7—C8—H8B	109.4	H16B—C16—H16C	109.5
H8A—C8—H8B	108.0		
			
C6—C1—C2—C3	0.6 (2)	C8—O1—C9—O3	−3.7 (2)
C1—C2—C3—C4	−0.9 (3)	C8—O1—C9—C10	178.78 (12)
C1—C2—C3—Br1	178.42 (12)	O3—C9—C10—C15	−40.3 (2)
C2—C3—C4—C5	0.5 (3)	O1—C9—C10—C15	137.23 (15)
Br1—C3—C4—C5	−178.80 (12)	O3—C9—C10—C11	139.03 (17)
C3—C4—C5—C6	0.2 (2)	O1—C9—C10—C11	−43.5 (2)
C2—C1—C6—C5	0.0 (2)	C15—C10—C11—C12	−0.6 (2)
C2—C1—C6—C7	−178.94 (14)	C9—C10—C11—C12	−179.87 (15)
C4—C5—C6—C1	−0.4 (2)	C15—C10—C11—C16	177.21 (16)
C4—C5—C6—C7	178.59 (15)	C9—C10—C11—C16	−2.1 (2)
C1—C6—C7—O2	−174.45 (15)	C10—C11—C12—C13	−1.3 (3)
C5—C6—C7—O2	6.6 (2)	C16—C11—C12—C13	−179.19 (17)
C1—C6—C7—C8	6.2 (2)	C11—C12—C13—C14	1.8 (3)
C5—C6—C7—C8	−172.78 (14)	C12—C13—C14—C15	−0.3 (3)
C9—O1—C8—C7	−75.92 (17)	C13—C14—C15—C10	−1.6 (3)
O2—C7—C8—O1	−0.4 (2)	C11—C10—C15—C14	2.0 (3)
C6—C7—C8—O1	178.89 (12)	C9—C10—C15—C14	−178.63 (15)

### Hydrogen-bond geometry (Å, °)

**Table:**

*Cg*1 and *Cg*2 are the centroids of the C1–C6 and C10–C15 rings, respectively.

**Table:**

*D*—H···*A*	*D*—H	H···*A*	*D*···*A*	*D*—H···*A*
C8—H8A···O2^i^	0.99	2.32	3.224 (2)	151
C8—H8B···O2^ii^	0.99	2.52	3.447 (3)	156
C15—H15A···Cg1^iii^	0.95	2.74	3.5472 (19)	143
C16—H16B···Cg1^iv^	0.98	2.98	3.4909 (19)	114
C2—H2A···Cg2^v^	0.95	2.91	3.5915 (19)	130

Symmetry codes: (i) *x*, −*y*+3/2, *z*+1/2; (ii) *x*−1, *y*, *z*; (iii) *x*−1, −*y*+1/2, *z*−3/2; (iv) *x*+1, −*y*+1/2, *z*−1/2; (v) *x*, −*y*+1/2, *z*−1/2.
